# Electrostatic interactions constrain generalization of porous-media models for intracellular diffusion in mammalian cells

**DOI:** 10.1073/pnas.2605166123

**Published:** 2026-04-10

**Authors:** Debabrata Dey, Gideon Schreiber

**Affiliations:** ^a^Amity Institute of Click Chemistry Research and Studies, Amity University, Noida, Uttar Pradesh 201313, India; ^b^Department of Biomolecular Sciences, Weizmann Institute of Science, Rehovot 7610001, Israel

Destrian et al. (1) present a unique combination of live cell fluorescence recovery after photobleaching (FRAP), fluorescence correlation spectroscopy (FCS) measurements and multiscale modeling to argue that cytoplasmic crowding can be described as a porous medium. The work is technically impressive and provides a useful physical framework for interpreting diffusion of inert tracers in cells.

However, the experimental conclusions rely primarily on green fluorescent protein (GFP) as a tracer, that is relatively inert and weakly interacting. In this regime, it is reasonable that steric hindrance, tortuosity, and hydrodynamic effects dominate, and the porous-medium description performs well. The broader cytoplasmic environment, however, is highly heterogeneous and strongly charged, and many proteins and small molecules experience substantial electrostatic and nonspecific interactions that can markedly alter their mobility.

**Fig. 1.**
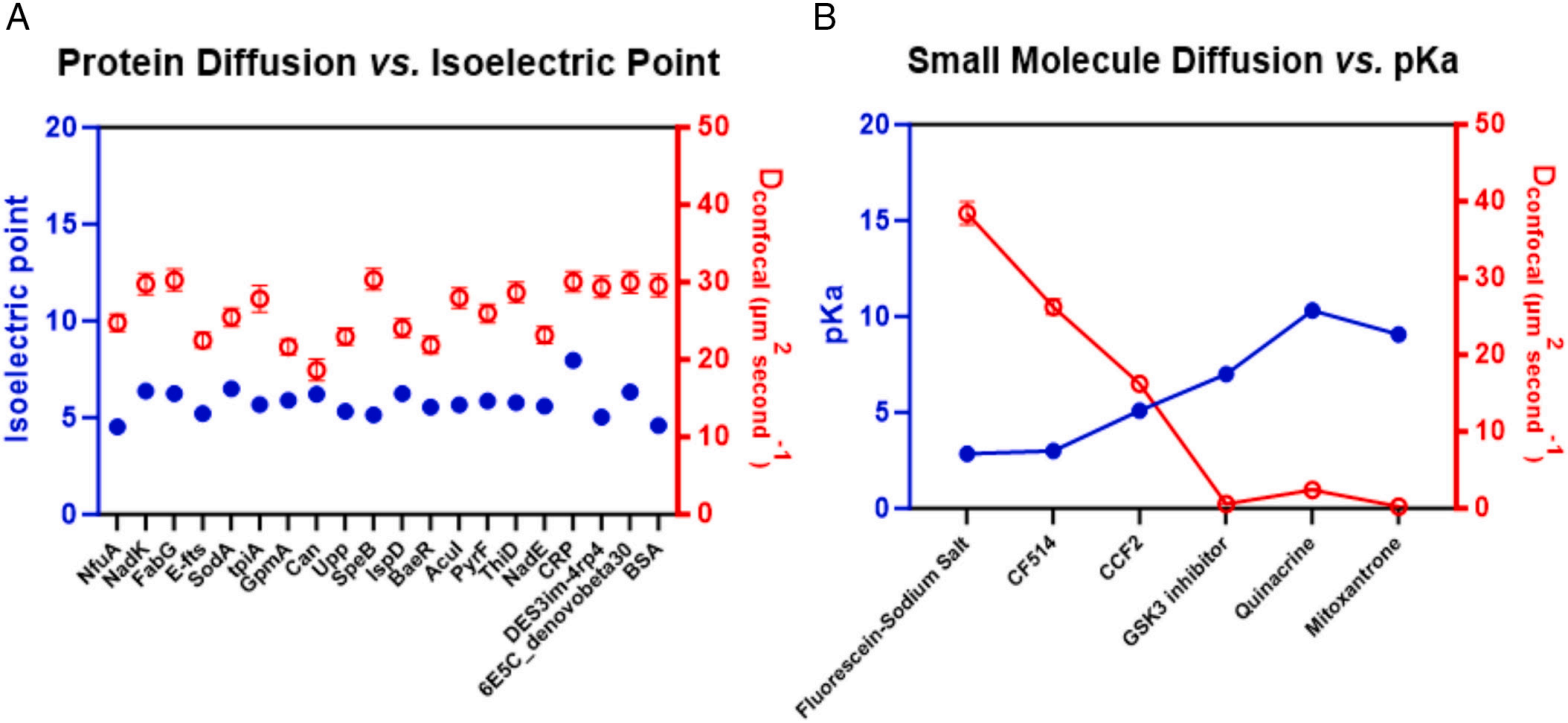
(*A*) Isoelectric point vs. D_confocal_ (experimentally measured diffusion coefficient values) for bacterial proteins and (*B*) Dependency of D_confocal_ values on p*K*a of small molecule drugs in HeLa cell cytoplasm are shown. Parts of panels *A* and *B* are adapted from Dey et al., eLife (2), 10.7554/eLife.97255.3, licensed under CC BY.

In our FRAP measurements of 18 bacterial proteins (Isoelectric point, pI ranges between 4.5 to 8, i.e., the majority are acidic) microinjected in HeLa cells, diffusion coefficients between ~18 to 30 μm^2^ s^−1^ were measured, (2) (Fig. 1*A*) consistent with the values reported for many inert proteins in mammalian cell cytoplasm (3, 4). Wild type GFP (PDB ID 1GFL) has a pI of 6.19 with a net charge of −5.53 at pH 7.0 (5). Previous experiments done in the cytosol of *Escherichia coli* have shown that introducing a small tag consisting of six histidine residues in GFP reduces diffusion coefficient by as much as 40%, highlighting the strong impact of charge-mediated interactions (6). Therefore, a single effective cytoplasmic viscosity/crowding effect cannot explain this reduction at least in bacterial cytosol. Estrada et al have shown that increasing the net negative charge of a protein reduces its retardation by clustering and accelerates rotational diffusion in living cells (7). In line with these observations, Schavemaker et al. systematically increased the positive surface charge of GFP variants in *E. coli* and two other prokaryotes and reported that positively charged proteins can diffuse up to two orders of magnitude more slowly than negatively charged counterparts (8). Compared to bacteria cytosol, mammalian cell cytoplasm is far more complex with the presence of many organelles including the acidic ones like lysosomes. Consistent with this, we found that weakly basic (p*K*a > 7), amine-containing small molecule drugs (average Molecular weight < 1 kDa) exhibit pronounced intracellular “stickiness,” (as supported by lower D_confocal_ values with increasing p*K*a) which is consistent with widespread nonspecific electrostatic interactions with anionic cellular components present in the cell cytoplasm as shown in Fig. 1*B* (2). Even when we tried to block the acidification of acidic chambers of cells, i.e., lysosomes, the overall diffusion did not increase much, which further supports the existence of nonspecific electrostatic interactions throughout the cell cytoplasm (2).

These observations suggest that while the porous-medium framework is highly appropriate for weakly interacting tracers such as GFP, its generalization to charged proteins especially positively charged macromolecules, signaling molecules, and drugs will likely require explicit consideration of electrostatic and other related nonspecific interactions. Incorporating these effects may be essential for extending the model to the full diversity of intracellular transport processes, particularly in dynamic or heterogeneous cellular states.
